# Editorial: Mechanisms and novel treatments of muscle wasting

**DOI:** 10.3389/fmed.2026.1814819

**Published:** 2026-03-19

**Authors:** Siqi Wang, Yifu Chen, Guangjie Cai, Sapana Kushwaha, Young Hoon Son, Jin Li, Junjie Xiao

**Affiliations:** 1 Shanghai Applied Radiation Institute, School of Environmental and Chemical Engineering, Shanghai University, Shanghai, China; 2 Cardiac Regeneration and Ageing Lab, Institute of Cardiovascular Sciences, Shanghai Engineering Research Center of Organ Repair, Joint International Research Laboratory of Biomaterials and Biotechnology in Organ Repair (Ministry of Education), School of Life Sciences, Shanghai University, Shanghai, China; 3 Department of Pharmacology and Toxicology, National Institute of Pharmaceutical Education and Research, Raebareli (NIPER-R), Lucknow, Uttar Pradesh, India; 4 Biohybrid Systems Group, Coulter Department of Biomedical Engineering, Georgia Institute of Technology, Emory University School of Medicine, Atlanta, GA, United States

**Keywords:** editorial, mechanism, muscle atrophy, muscle wasting, treatment

Muscle atrophy is a pathological condition triggered by various factors such as nerve injury and prolonged immobilization, characterized by a reduction in skeletal muscle volume, diminished muscle strength, and impaired motor function. As it can trigger multiple complications and increase health risks and the burden on healthcare services, elucidating its pathogenesis and exploring effective interventions has become a significant research priority (1–4).

In this Research Topic, we highlight eight recent studies on muscle atrophy, covering therapeutic interventions, diagnostic biomarkers, and potential molecular mechanisms. The contributions span diverse research designs, including randomized controlled trials (RCTs), meta-analyses, case-control studies and bioinformatics approaches. Together, these works provide valuable insights into the multifactorial pathogenesis of muscle atrophy and suggest new directions for clinical management and future research.

Sarcopenia is a progressive age-related condition characterized by loss of muscle mass, reduced strength, and functional decline. Although aging is the main contributing factor, chronic diseases and physical inactivity also accelerate its development (5, 6). Investigations into effective interventions for sarcopenia have yielded promising results across multiple approaches, reflecting a shift toward personalized and multi-targeted strategies. Otzel et al. evaluated hormone therapy in specific populations: a randomized, double-blind, placebo-controlled pilot study showed that high-dose testosterone replacement therapy (TRT) combined with finasteride (TRT + finasteride) effectively improves body composition and musculoskeletal health in male patients with chronic incomplete spinal cord injury. This combination therapy not only increases total lean body mass by 3%−4% and knee extensor cross-sectional area by 8%-11% but also reduces visceral fat mass by 9%−14% without inducing prostate enlargement, thereby addressing a key safety concern associated with traditional TRT.

Traditional Chinese Medicine (TCM) and acupuncture are increasingly investigated as adjunctive approaches for sarcopenia management. A systematic review and meta-analysis of clinical studies by Zhang et al. reported that specific TCM interventions (including TCM compound formulas, single-herb extracts, and massage) were associated with improvements in muscle mass, grip strength, muscle morphology, and glycemic control. Mechanically, the interventions activated the IGF-1-PI3K-Akt-mTOR pathway and inhibited FoxO/ubiquitin-proteasome degradation pathway. They also reduced inflammation and oxidative stress, improved insulin sensitivity, and supported mitochondrial integrity. Additionally, TCM exhibits bidirectional regulation of body weight (increasing weight in diabetic cachexia models and reducing weight in obesity-associated sarcopenia models). Similarly, Niu et al. conducted a meta-analysis of 10 RCTs and found that compared to conventional treatment, acupuncture significantly improved overall response rate, muscle mass, grip strength, and usual gait speed. In addition, acupuncture was associated with a reduction C-reactive protein levels. These results suggest that acupuncture may help improve muscle mass and physical functions in patients with sarcopenia and serve as a complementary option for frail older adults who cannot tolerate conventional treatments. However, the limited sample sizes and inconsistent methodological quality should be interpreted with caution. Larger, well-designed studies are necessary to confirm the benefits in clinical practice.

Novel therapeutic targets are also being explored. Jeong et al. reported that deferoxamine (DFO), an iron chelator, may attenuate glucocorticoid-induced muscle atrophy. DFO reduced the expression of atrophy related genes Atrogin-1 and MuRF1 by restoring intracellular iron homeostasis, enhancing AKT phosphorylation, inhibiting FOXO3a nuclear translocation, and reducing KLF15 expression. It simultaneously restores the IGF-1 and myostatin levels altered by dexamethasone treatment. DFO preserved the morphology of C2C12 myotube and maintained grip strength, tibialis anterior muscle mass and myofiber size in mouse models. These findings suggest that DFO mitigates glucocorticoid-induced muscle atrophy through modulation of the AKT/FOXO3a and KLF15 signaling pathways. Lu et al. highlighted lipid peroxidation as a key pathological driver of sarcopenia. Lipid peroxidation accelerates muscle loss and functional decline by damaging cell membranes, promoting mitochondrial dysfunction, and inducing abnormal muscle cell death, processes that influenced by genetic susceptibility and chronic diseases. The authors proposed that GPX4, a lipid lipid peroxide scavenging enzyme, and n-3 polyunsaturated fatty acids may protect skeletal muscle by inhibiting oxidative damage. They also discussed a variety of potential interventions, including natural compounds (e.g., astaxanthin and quercetin), nutritional supplements (e.g., vitamin E and glutamine), plant-derived extracts (e.g., Schisandra extract and lemon peel extracts), and lifestyle interventions such as exercise training and electrical stimulation. Collectively, these strategies may slow sarcopenia progression through antioxidant and metabolic regulation, supporting a multi-dimensional strategy for prevention and treatment.

Accurate and early diagnosis is crucial for the timely intervention in sarcopenia. Liu et al. advanced this field by identifying potential biomarkers and screening tools. In a case-control study of 343 participants aged 65 and over, including 49 individuals diagnosed with sarcopenia according to AWGS criteria (defined by reduced grip strength and low appendicular skeletal muscle mass), circulating cathepsin K (CatK) emerging as a promising diagnostic marker: Serum CatK levels were significantly elevated in sarcopenia patients, and were negatively correlated with grip strength. The diagnostic performance of and CatK yielded an area under the curve (AUC) of 0.704, outperforming cathepsin D. These finding suggest that circulating CatK may serve as a simple and cost-effective screening biomarker, particularly in resource-limited clinical settings.

Diagnostic approaches have been further enhanced through bioinformatics and machine learning. Wang et al. using the GSE226151 dataset, identified three signature genes (CXCR1, CXCR2, and LPL) linked in chemokines signaling and neutrophil extracellular trap formation (NETosis). Their nomogram model showed strong diagnostic performance in distinguishing sarcopenia from healthy controls (AUC = 0.837) and pre-sarcopenic states (AUC = 0.903), highlighting a novel molecular diagnostic approach for early detection. Additionally, Li et al. conducted a multicenter case-control study among hospitalized elderly individuals aged 65 and above to investigate the association between cardiovascular health metrics and sarcopenia. They evaluated the American Heart Association's Life's Essential 8 (LE8) and the extended Life's Crucial 9 (LC9), which incorporates mental health assessment via the PHQ-9. LC9 demonstrated superior predictive value compared with LE8, with higher scores correlating with greater muscle mass and strength. Together, these finding support an integrated screening strategy that captures both cardiometabolic and psychosocial dimensions of sarcopenia risk.

Recent studies have improved our understanding of the mechanisms underlying sarcopenia, highlighting the important roles of inflammation, metabolic disorders and signaling pathway dysfunction. Chronic inflammation has been identified as a key driver of muscle wasting. Evidence suggests that chemokine-mediated NETosis induces muscle atrophy by activating the JAK-STAT signaling pathway and cytokine-receptor interactions, with CXCR1 and CXCR2 identified as important mediators. These findings support the potential of acupuncture and TCM to alleviate sarcopenia, in part through regulation of inflammatory responses and improvement of the muscle microenvironment.

The abnormal mechanisms within metabolic and signaling pathways have also been elucidated: diabetic sarcopenia is associated with insulin resistance, oxidative stress, and mitochondrial dysfunction. TCM based interventions may help restore protein homeostasis by activating the IGF1-PI3K-Akt-mTOR anabolic pathway while suppressing FoxO/ubiquitin-proteasome degradation. In spinal cord injury related sarcopenia, low testosterone levels can worsen muscle loss and bone decline, whereas, testosterone replacement therapy combined with finasteride may normalize hormone levels improve muscle and bone health. Furthermore, iron overload has been linked to glucocorticoid-induced muscle atrophy through disruption of AKT-FOXO3a signaling, emphasizing the important interplay between metal metabolism and muscle function.

The eight studies summarized here provide a comprehensive overview of the pathophysiology, therapeutics strategies, and emerging diagnostic approaches for sarcopenia (7, 8). The field of sarcopenia therapeutics continues to evolve, ranging fom hormonal and TCM interventions to novel pharmacological targets. Meanwhile, advances in biomarkers and risk stratification tools are improving early detection and enabling personalized management. Nevertheless, important challenges remain, including the limited sample sizes and significant methodological heterogeneity between acupuncture and TCM research. In addition, longitudinal studies are needed to clarify causal relationships between candidate biomarkers and sarcopenia progression.
